# A Classical Case of Sessile Osteochondroma in a 13-Year-Old Boy

**DOI:** 10.7759/cureus.67189

**Published:** 2024-08-19

**Authors:** Azeem I Saifi, Pratapsingh Parihar, Iram Saifi, Khizer Ansari

**Affiliations:** 1 Medicine and Surgery, Jawaharlal Nehru Medical College, Datta Meghe Institute of Higher Education and Research, Wardha, IND; 2 Radiodiagnosis, Jawaharlal Nehru Medical College, Datta Meghe Institute of Higher Education and Research, Wardha, IND; 3 Radiology, Jawaharlal Nehru Medical College, Datta Meghe Institute of Higher Education and Research, Wardha, IND; 4 Medicine, Jawaharlal Nehru Medical College, Datta Meghe Institute of Higher Education and Research, Wardha, IND

**Keywords:** bone tumors, osteoid osteoma, fibrous dysplasia (fd), aneurysmal bone cyst, eosinophilic granuloma, sessile osteochondroma

## Abstract

This case report highlights the understanding of the swelling feature of the right knee in a young adult and gives an overview of bone tumors. We are presenting a case of right knee swelling in a 13-year-old boy who was anxious before the investigations. Additionally, this report provides an approach for an accurate diagnosis of swelling. It highlights the approach to bone swelling and provides an overview of how to classify bone tumors. With the help of advanced technology such as magnetic resonance imaging (MRI), it gives detailed information about the nature of bone tumors, especially in the case of sessile osteochondroma. This case report also gives us information about the classification of bone tumors and their progression and guides us toward management with the help of an MRI.

## Introduction

The systematic approach to diagnosing any bone tumor depends on the age of the tumor and its location, whether it is epiphysis, metaphysis, or diaphysis. The location should also mention the centricity of the tumor, whether it is centric, eccentric, or juxtacortical. The next thing to notice is the presence of a zone of transition, periosteal reaction, matrix, and the number of lesions.

To diagnose any bone tumor on a radiograph, it should be characterized based on age and the lytic/sclerotic type of lesion. The location of the tumor narrows down the further diagnostic approach. Most of the time, metaphyseal lesions can reach into diaphysis, or diaphyseal lesions can also extend into metaphysis.

Based on age, tumors can be divided into less than 30 years and more than 30 years of age. Less than 30 years include eosinophilic granuloma, osteochondroma, simple bone cyst, aneurysmal bone cyst, non-ossifying fibroma, and fibrous dysplasia. For more than 30 years, it is important to consider metastasis, multiple myeloma, chondromyxoid fibroma, and enchondroma [1].

Based on location, epiphyseal lesions include giant cell tumors and chondroblastoma. Metaphyseal lesions include osteochondroma, simple bone cyst, aneurysmal bone cyst, non-ossifying fibroma, enchondroma, and osteosarcoma. Diaphyseal lesions include osteoid osteoma and Ewing sarcoma. Benign tumors usually have well-defined margins, a narrow zone of transition, and a solid type of periosteal reaction such as osteoid osteoma, while malignant tumors are usually ill-defined, have a wide zone of transition, and can have a lamellated, spiculated, or Codman's type of periosteal reaction. Ewing sarcoma shows a lamellated type of reaction; osteosarcoma shows Codman's triangle and a sunburst type of reaction. Some benign tumors do not show any type of periosteal reaction, such as fibrous dysplasia, enchondroma, non-ossifying fibroma, and simple bone cysts [2].

Cortical destruction can also help to distinguish between benign and malignant tumors. For instance, irregular cortical destruction is commonly observed in osteosarcoma and locally aggressive tumors such as Ewing sarcoma. Other types of cortical destruction include ballooning, which causes endosteal destruction of the cortex, and new bone formation on the outside, resulting in expansion and the production of expansile lesions such as giant cell tumors and chondromyxoid fibroma. The next important thing to distinguish is the matrix. Chondroid matrix is found in chondrosarcomas and enchondromas, while osteoid matrix can be seen in osteosarcoma and osteoid osteoma.

## Case presentation

We present the case of a 13-year-old male who presented with swelling in his right knee for six months, associated with pain and discomfort while walking. There was no history of weight loss, trauma, or fever, and there are no comorbidities such as hypertension, diabetes mellitus, tuberculosis, or any other chronic illness. The patient had no history of neonatal intensive care unit stays or low birth weight. All milestones were achieved within the normal time duration. There is no history of any developmental delays.

On examination, there is no cyanosis, icterus, pallor, clubbing, or lymphadenopathy. Pulse was 82 beats/min, and blood pressure was 110/82 mmHg. On palpation, there was a hard palpable lump approximately 7 x 5 cm on the medial aspect of the right distal thigh. There is no increase in temperature or tenderness. On inspection, there was no other swelling found on the bilateral lower limbs, upper limbs, spine, ribs, or skull.

The cardiovascular, respiratory, central nervous, and genitourinary systems were within normal limits. The patient was advised to get an X-ray of the right knee.

**Figure 1:**
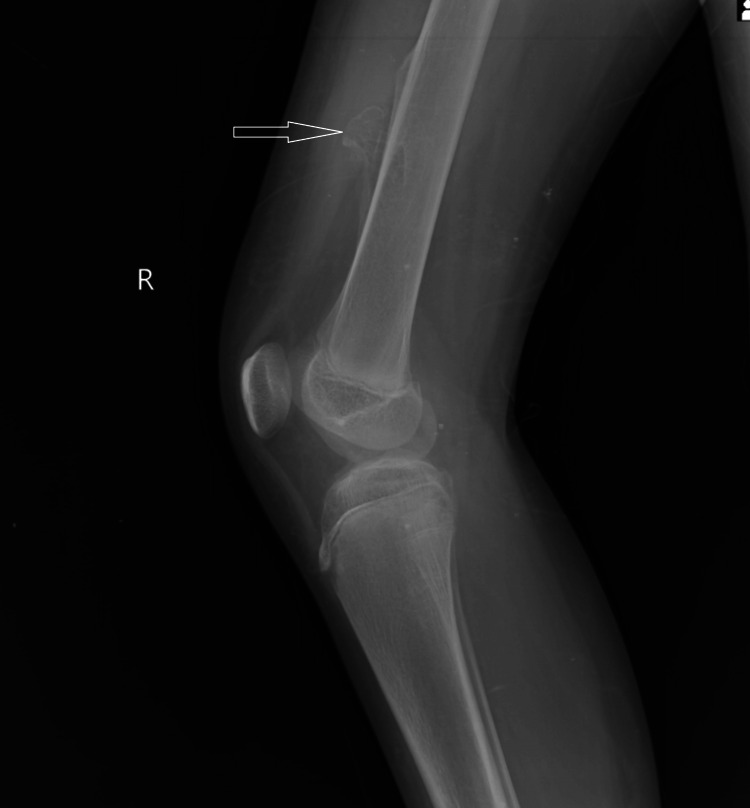
The lateral view radiograph of the right knee with the distal femur and the proximal tibia in an immature skeleton shows a sessile bony outgrowth in the meta-diaphyseal location of the distal femur.

**Figure 2:**
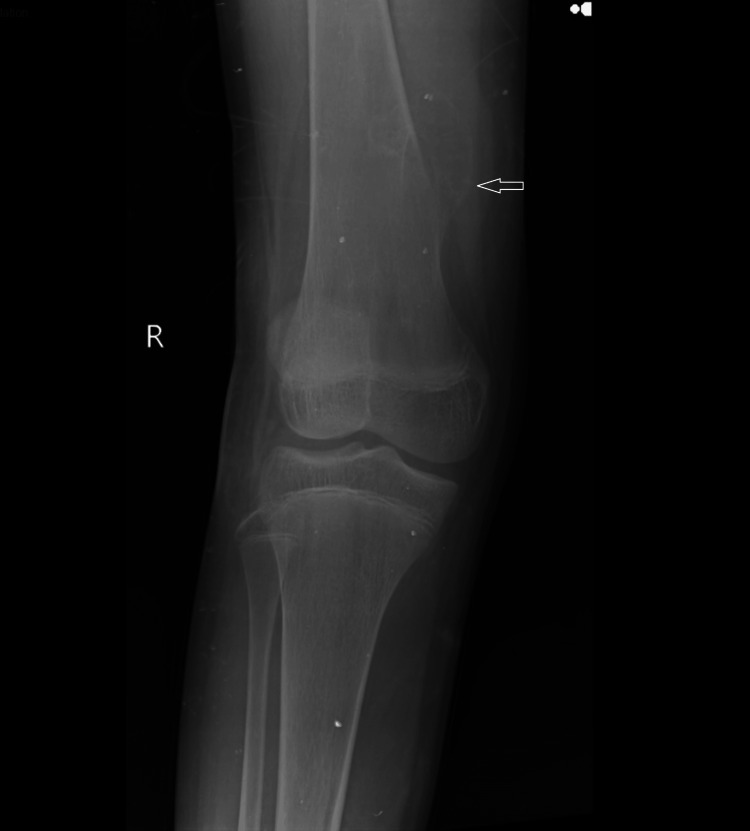
The AP view radiograph of the right knee with the distal femur and the proximal tibia in an immature skeleton shows a sessile bony outgrowth in the meta-diaphyseal location of the distal femur on the medial side. AP: anteroposterior

The frontal and lateral radiographs of the right knee with the distal femur and proximal tibia and fibula in an immature skeleton show a well-defined, sessile outgrowth in the distal meta-diaphyseal region not involving the knee joint with an associated broadening of the distal meta-diaphysis of the right femur (Figures 1, 2). The tibia, fibula, and patella appear normal, with no evidence of a fracture or any other bony abnormality noted.

**Figure 3:**
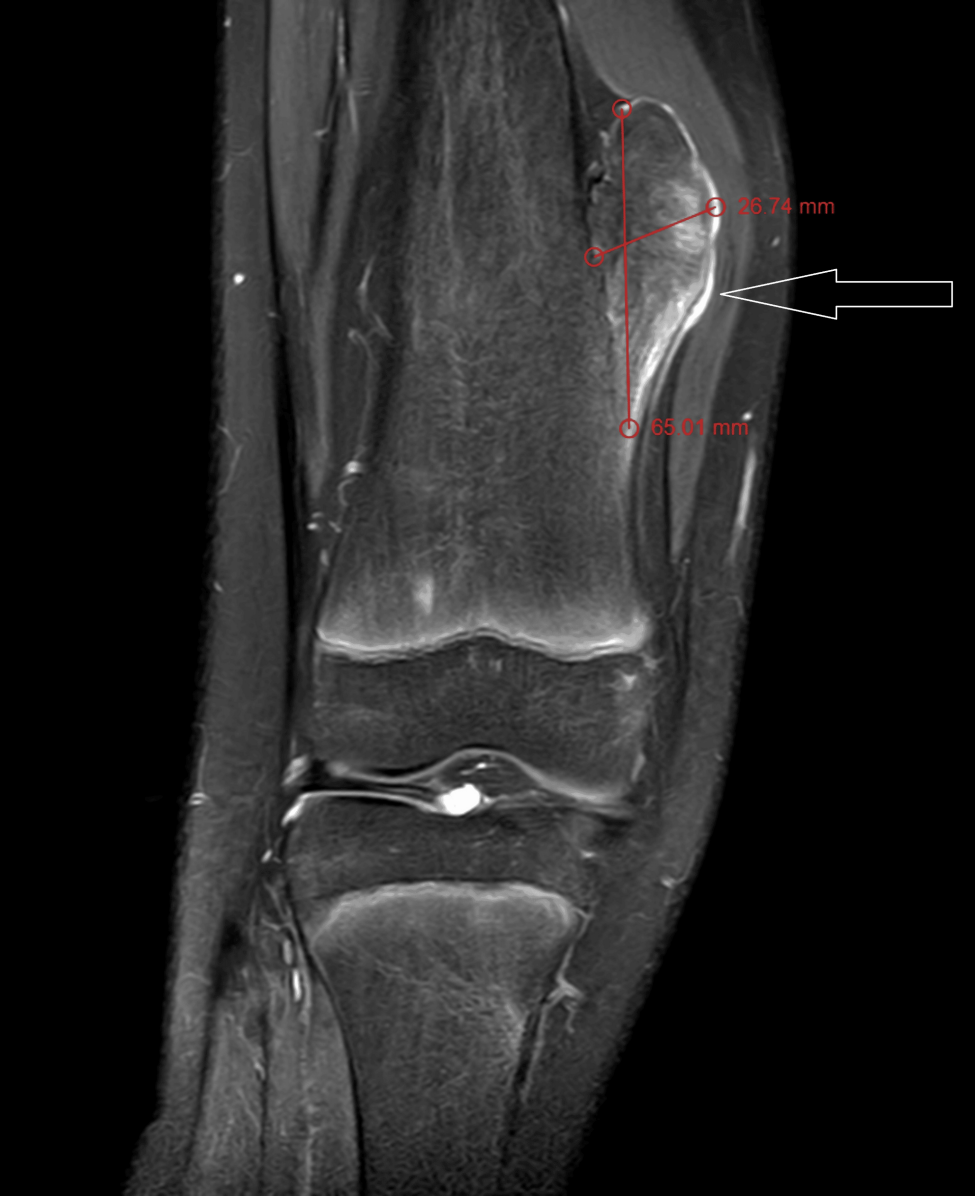
The coronal T1 fat-suppressed post-contrast image shows a non-enhancing, sessile bony outgrowth arising from the distal meta-diaphyseal region of the medial aspect of the right knee, measuring 65.01 × 26.74 × 40.48 mm. There is no evidence of enhancement and involvement of the surrounding soft tissue.

**Figure 4:**
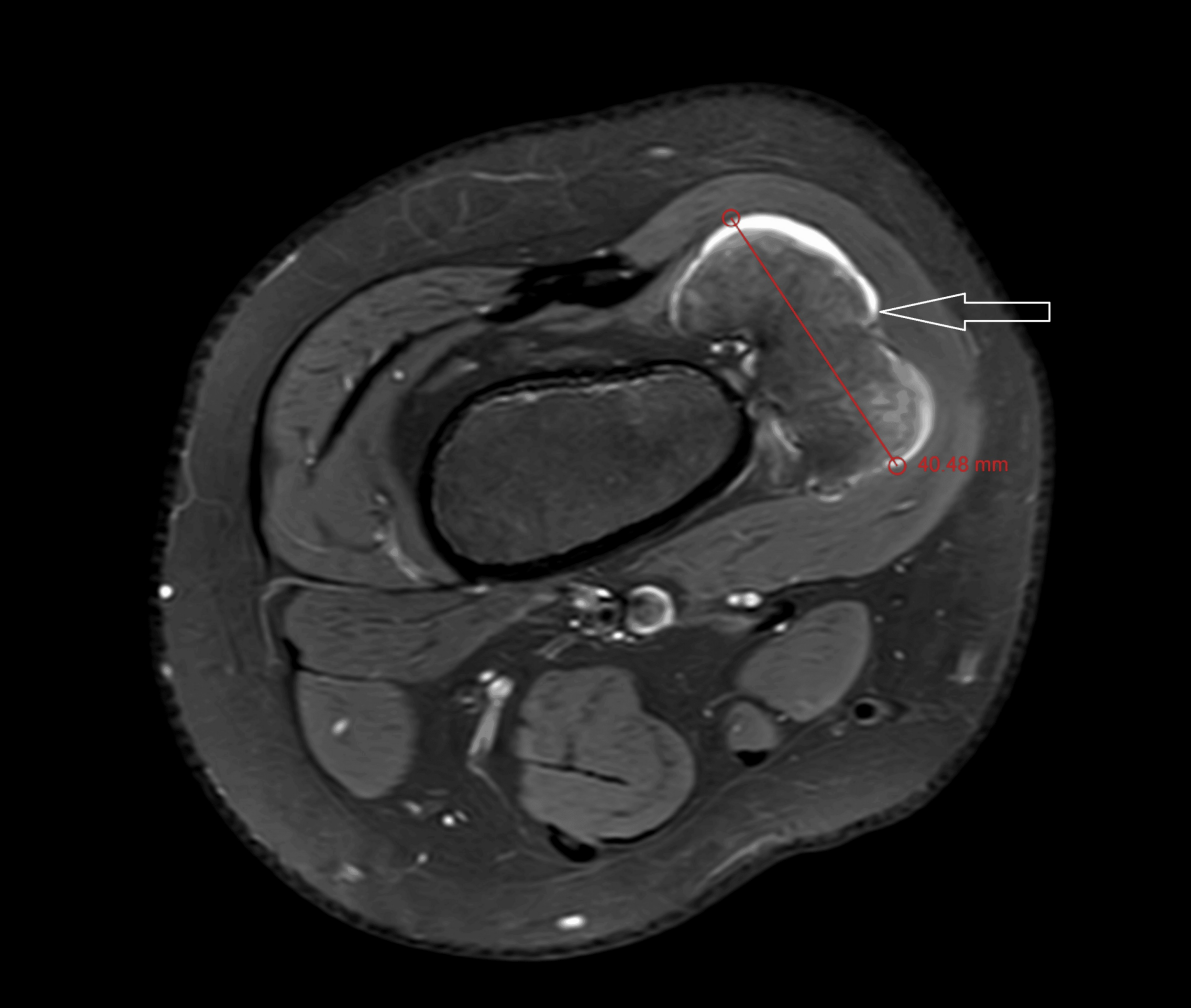
The axial PD fat-suppressed MRI shows the anteroposterior dimension of the sessile outgrowth and the continuation with the parent bone. PD fat sat: proton density fat suppression image

**Figure 5:**
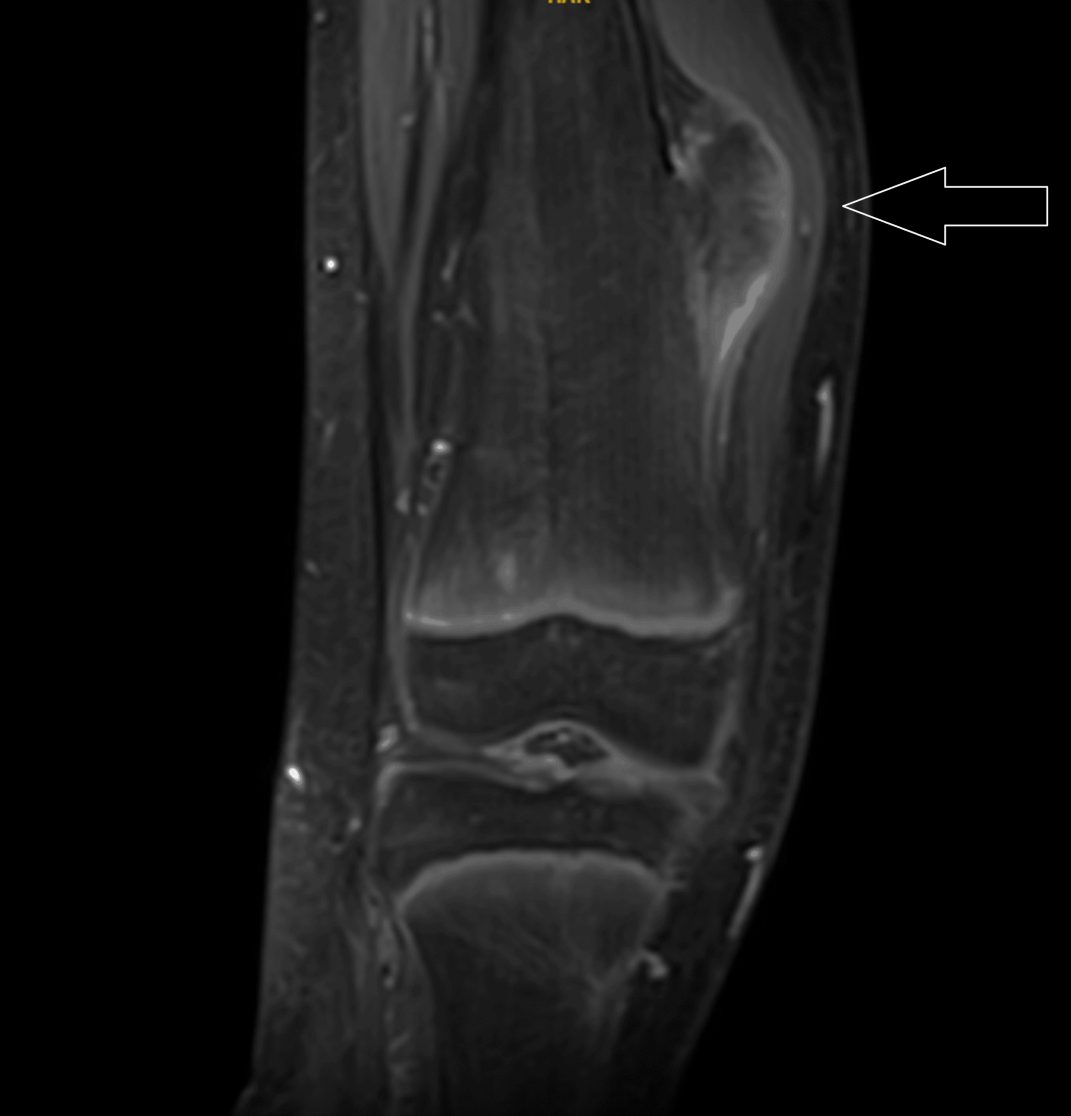
The coronal PD fat sat image indicates that the lesion is causing splaying and compression of the adjacent vastus medialis muscle. PD fat sat: proton density fat suppression image

**Figure 6:**
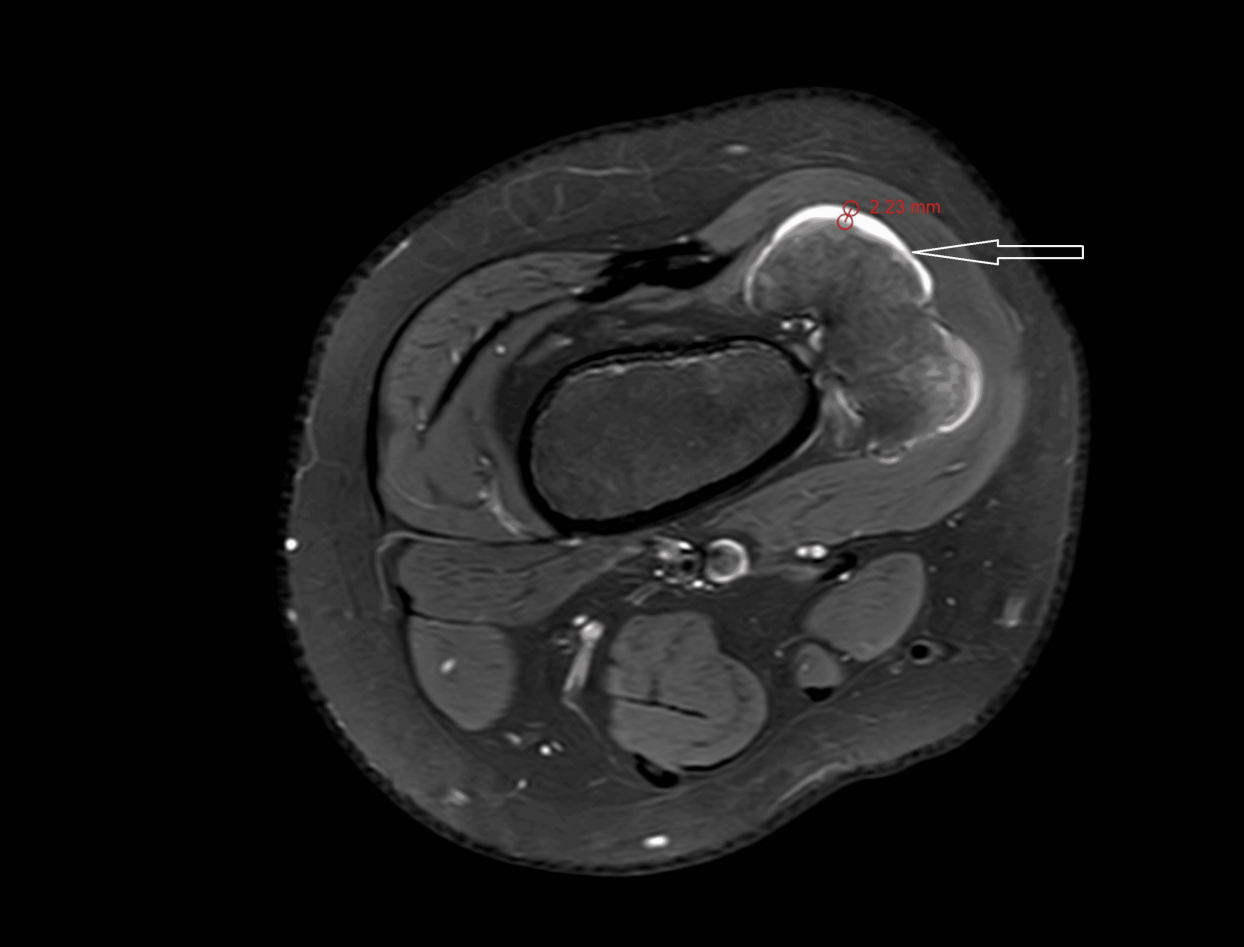
The PD fat sat axial image shows that the cartilage cap measures 2.23 mm in thickness. PD fat sat: proton density fat suppression image

**Figure 7:**
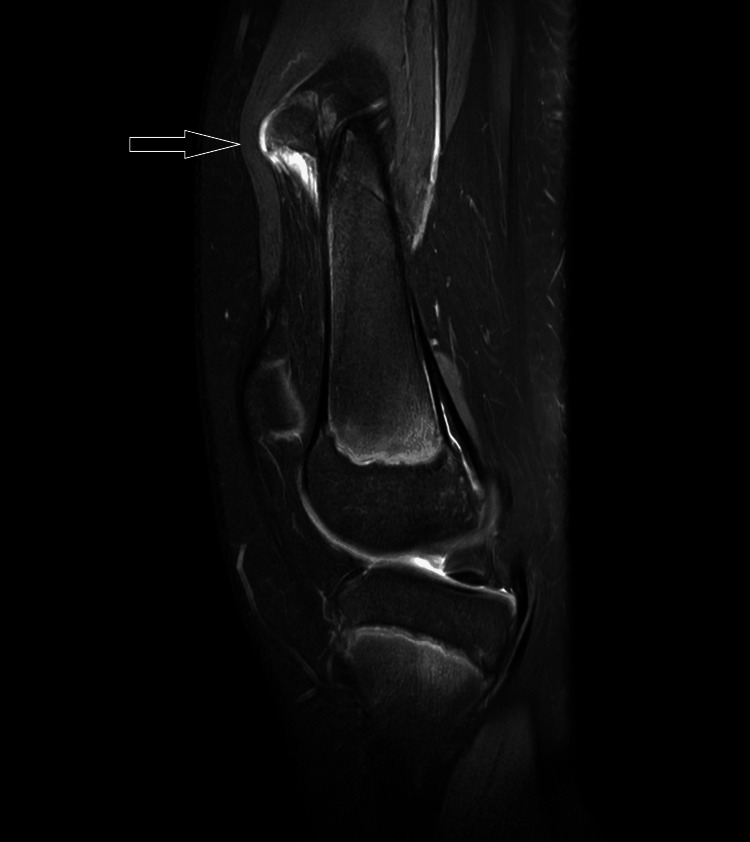
The sagittal PD fat sat image shows that the lesion is causing splaying and compression of the adjacent vastus medialis muscle. PD fat sat: proton density fat suppression image

**Figure 8:**
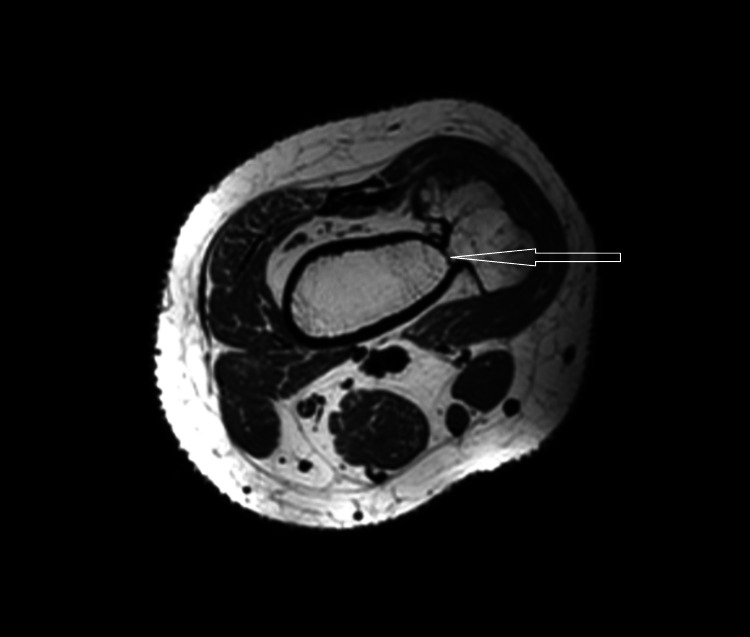
The axial T1WI image shows the continuity of the lesion with the cortex and medullary cavity of the parent femoral bone. T1W1: T1-weighted imaging

MRI T1 contrast shows a non-enhancing sessile bony outgrowth arising from the distal meta-diaphyseal region of the medial aspect of the right knee measuring 65.01 × 26.74 × 40.48 mm (Figures 3, 4). The axial proton density (PD) fat suppression images show continuity with the cortex and medullary cavity of the parent femoral bone (Figures 4, 8). On PD fat suppression axial images, the cartilage cap measures 2.23 mm in thickness (Figure 6). On coronal and sagittal PD fat-saturated images, the lesion is causing splaying and compression of the adjacent vastus medialis muscle (Figures 5, 7). There is no evidence of enhancement or involvement of the surrounding soft tissue (Figure 3).

**Figure 9:**
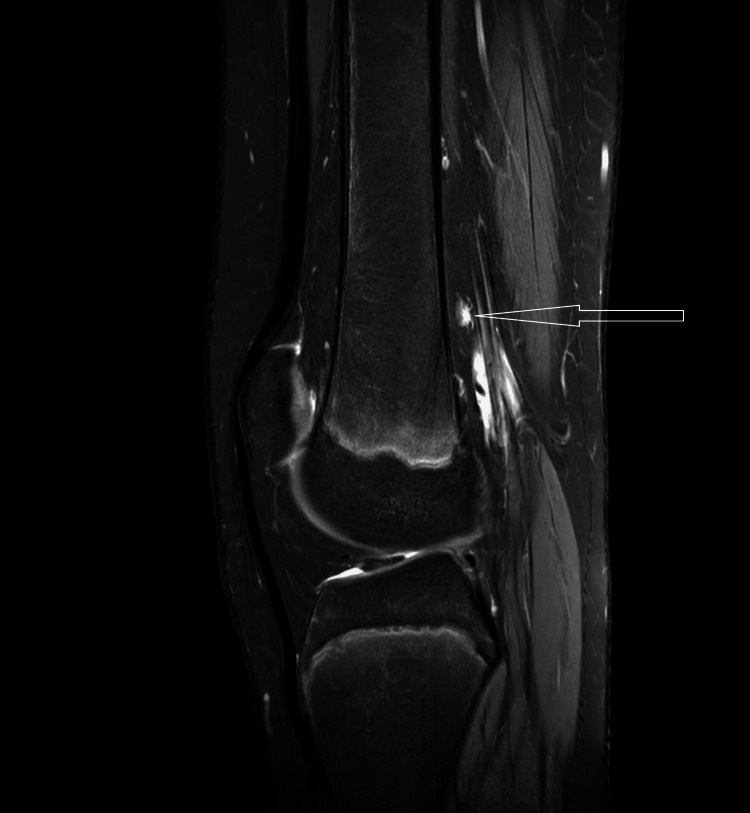
The sagittal PD fat sat image shows the presence of a popliteal lymph node. PD fat sat: proton density fat suppression image

All the ligaments are normal. The anterior and posterior cruciate ligaments exhibit normal signal intensity and morphology. The collateral ligament and menisci appear normal. The proximal tibia, fibula, and patella appear normal without evidence of any fracture lines. A subcentimetric popliteal lymph node was noted (Figure 9). The patient's MRI revealed a diagnosis of sessile osteochondroma.

A limb-sparing procedure using local excision was performed to address both cosmetic concerns and alleviate discomfort while walking. The patient was prescribed analgesic diclofenac 50 mg twice a day, pantaprozole 40 mg once a day, and antibiotic cefixime 200 mg twice a day for seven days after surgery. The patient was instructed to observe bed rest for a duration of one week and schedule a follow-up appointment after 15 days. The surgery yielded a highly effective response after 15 days. The patient was mobile, and there was no discomfort while walking. The patient was also instructed to undergo a follow-up MRI in the event of recurring swelling or pain when walking, in order to assess the treatment response and detect any residual tumor or potential malignant transformation.

## Discussion

Osteochondroma is a benign tumor predominantly found in young adults and predominantly in meta-diaphyseal locations. There are usually two types: sessile and pedunculated. Pedunculated osteochondroma has a narrow base, while sessile osteochondroma has a broad base. Osteochondromas can be solitary or multiple. The multiple form is autosomal-dominant, referred to as hereditary multiple exostosis (HME) or familial osteochondromatosis. Cartilage caps are found in both solitary and HME, surrounding the periosteum. Another way to differentiate it from exostosis is that exostosis is usually multiple.

Osteochondroma can be primary or secondary. In primary osteochondroma, no cause is found, but secondary osteochondroma is found due to post-radiation, post-surgery, and Salter-Harris fractures [3,4]. For the diagnosis of osteochondroma, it is important to communicate the tumor stalk with the medulla and cortex of the parent bone. Another characteristic feature is the presence of a cartilage cap and the growth of a pedunculated tumor away from the joint until the fusion of the epiphysis occurs. These growths possess a chondroid matrix and are predominantly located in the lower extremities. The femur is the most frequent site in the lower limb. The humerus is the most frequent site in the upper limb. They can be found in the hands and also in the scapula [5,6]. Sometimes they can also be seen in the spine, predominantly in the posterior elements and in the ribs. If found in the ribs, it can create dangerous complications such as osseous deformation, fracture, bursa formation, vascular compromise, neurological symptoms, and malignant transformation. Growth and change in morphology after skeletal maturation are suspicious features of pneumothorax and hemothorax [7,8]. The diagnosis of osteochondroma requires a plane radiograph, which can differentiate whether it is sessile or pedunculated and can also depict the location of the tumor. Computed tomography can give extensive detail of the tumor and the communication of medullary continuity.

MRI plays a very important role in categorizing the tumor as benign or malignant. For the demonstration of the cartilaginous cap, ultrasound and MRI play a vital role. Cartilage thickness is very important to assess the malignant transformation of tumors. A cartilage thickness of up to 3 cm can be considered benign in younger patients; however, in adults, a cartilage cap exceeding 1.5 cm after skeletal maturation might also raise suspicion of malignancy [3,7]. In ultrasound, cartilage appears hypoechoic and superficial to the bone. In MRI, cartilage shows an intermediate to low signal on T1 and appears hyperintense on T2. Following the administration of contrast, benign tumors may not exhibit enhanced cartilage, although the surrounding soft tissues may exhibit enhancement [9]. If there is involvement of adjacent soft tissues, nerves, and vessels, the presence of bony edema favors malignant transformation.

Osteochondromas are tumors that occur near growth plates. Treatment depends on the age of the diagnosis because osteochondromas usually stop growing after skeletal maturity. Benign tumors can be advised for follow-up to see any changes for malignant transformation. They can be treated surgically due to the restriction of movement and for cosmetic reasons. Surgical indications of solitary and multiple osteochondromatosis are symptomatic lesions, aesthetically displeasing lesions, or lesions with suspicious imaging features such as irregular or indistinct margins, focal areas of radiolucency, bony erosions, or destruction. In spinal osteochondromas, surgical excision is an indication in radiculopathy, myelopathy, or vascular compression cases. If malignant transformation is suspected, wide local excisions should be performed. However, recurrence can occur in both benign and malignant lesions due to the presence of residual cartilage cells at the excision site following surgery. Postoperative monitoring is necessary to detect any potential relapse [3,10].

## Conclusions

This case report emphasizes the importance of diagnosing bone tumors and helps in distinguishing between benign and malignant lesions. Furthermore, it places significant importance on the patient's age when diagnosing bone cancer. Diagnosis by advanced technology such as MRI not only helps to differentiate the benign and malignant nature of the tumor but also helps to see the invasion of soft tissue structures and guide us for better management of the tumor. This case report contributes to the diagnosis and management of osteochondroma. Utilizing new technologies to confirm a diagnosis alleviates the anxiety experienced by both the patient and their guardian, thereby facilitating more effective management.
